# Understanding Treatment Patterns and Outcomes among Patients with De Novo Unresectable Locally Advanced or Metastatic Urothelial Cancer: A Population-Level Retrospective Analysis from Alberta, Canada

**DOI:** 10.3390/curroncol29100599

**Published:** 2022-10-12

**Authors:** Nimira Alimohamed, Simrun Grewal, Heidi S. Wirtz, Zsolt Hepp, Stephanie Sauvageau, Devon J. Boyne, Darren R. Brenner, Winson Y. Cheung, Tamer N. Jarada

**Affiliations:** 1 Tom Baker Cancer Centre, University of Calgary, Calgary, AB T2N 4N2, Canada; 2 Seagen Inc., Bothell, WA 98021, USA; 3 Seagen Canada, Mississauga, ON L5N 1W1, Canada; 4 Oncology Outcomes (O2), Calgary, AB T2N 4N2, Canada

**Keywords:** urothelial carcinoma, Canada, real-world data, treatment patterns

## Abstract

Despite a high disease burden, real-world data on treatment patterns in patients with unresectable locally advanced or metastatic urothelial carcinoma (la/mUC) in Canada are limited. This retrospective, longitudinal cohort study describes treatment patterns and survival in a population of patients with de novo unresectable la/mUC from Alberta, Canada, diagnosed between 1 January 2015 and 31 December 2019, followed until mid-2020. The outcomes of interest were systemic therapy treatment patterns and overall survival (OS). Of 206 patients, most (65.0%, *n* = 134) did not receive any systemic therapies. Of 72 patients (35.0%) who received first-line systemic therapy, the median duration of treatment was 2.8 months (IQR 3.3). Thirty-five patients (48.6% of those who received first-line therapy) received subsequent second-line therapy, for a median of 3.0 months (IQR 3.3). In all patients (*n* = 206), the median OS from diagnosis was 5.3 months (95% CI, 4.5–7.0). In patients who received treatment, the median OS from the initiation of first-line and second-line systemic therapy was 9.1 (6.4–11.6) and 4.6 months (3.9–19.2), respectively. The majority of patients did not receive first-line systemic therapy, and, in those who did, survival outcomes were poor. This study highlights the significant unmet need for safe and efficacious therapies for patients with la/mUC in Canada.

## 1. Introduction

In Canada, bladder cancer is the fifth most common cancer among adults, with approximately 12,500 new cases diagnosed in 2021 (9500 of these in men) and a 5-year survival rate of 77% [1]. Globally, approximately 95% of bladder cancers are urothelial carcinomas (UC), the most common histological form, with the incidence of UC believed to be increasing as a consequence of population growth and aging [2,3]. Although most cases of UC at diagnosis are superficial or non-muscle invasive (NMIBC), around 11% of patients with UC are diagnosed with locally advanced or metastatic disease (la/mUC) [4].

UC is considered unresectable locally advanced when cancer cells have spread outside the bladder wall to the adjacent pelvic or abdominal wall, and metastatic (N2, N3, M1, M1b) when cancer cells have spread into distant lymph nodes and/or distant organs, and require treatment with systemic chemotherapy [4,5,6,7]. Patients with unresectable or metastatic UC can be treated with systemic chemotherapy with the goal of delaying the time to progression and improving survival [4,5,6,7]. Currently, the prognosis for patients with la/mUC is poor, with only 6.4% of patients with metastatic disease surviving beyond 5 years [8]. Platinum-based combination chemotherapy remains the standard of care for first-line treatment of patients with la/mUC; however, many patients do not receive this treatment; possibly being unwilling or unable to tolerate chemotherapy [9]. For example, in the USA, approximately 23–52% of patients with mUC do not receive first-line treatment, and among those who do, approximately 51% of patients are not eligible to receive the initial standard of care of cisplatin-based chemotherapy [10,11]. However, there are limited data for Canadian patients with la/mUC related to treatment patterns overall or on the proportions of patients who do not receive first-line therapy.

In Canada, the PD-L1 inhibitor, avelumab, approved in 2020, is recommended as maintenance therapy in those patients with la/mUC whose disease has not progressed on first-line platinum-based chemotherapy [12,13]. For patients who progress during or after first-line platinum-containing chemotherapy, current Canadian treatment guidelines recommend PD-1/L1 inhibitor immunotherapy with pembrolizumab (approved in 2018) [13,14]. More recently, enfortumab vedotin was approved in 2021 for patients who had previously received PD-1/L1 inhibitor and platinum-containing chemotherapy [15]. Erdafitinib, an oral fibroblast growth factor receptor inhibitor, received conditional approval in 2020 for patients with susceptible fibroblast growth factor receptor 3 or 2 genetic alterations, and whose disease has progressed during or following a prior line of chemotherapy [16].

In light of the changing treatment landscape for bladder cancer, there is a need for real-world evidence of treatment patterns and clinical outcomes in patients with la/mUC in Canada. The objective of this study was to describe the treatment patterns and overall survival (OS) in patients with de novo la/mUC in Alberta, Canada.

## 2. Materials and Methods

### 2.1. Study Design and Data Sources

This was a retrospective, longitudinal cohort study that included all patients diagnosed with de novo la/mUC within the province of Alberta, Canada, between 1 January 2015 and 31 December 2019. Patients were followed until mid-2020. De novo in this analysis refers to patients who presented with la/mUC at the time of initial diagnosis. The analysis was limited to de novo cases, as the registry databases capture cases at their first entry; as a result, there is no reliable method to ascertain recurrent or relapsed cases. De-identified patient data were acquired from the following databases: Alberta Cancer Registry (all patients diagnosed with cancer within the province of Alberta: ~200,000+ cases), the ARIA database (covering 17 provincial cancer centers and 4.5 million residents of Alberta), Discharge Abstract Database (inpatient hospitalizations), National Ambulatory Care Reporting System Database (inpatient and outpatient encounters with ambulatory care services), Practitioner Claims Database, and Pharmaceutical Information Network Database (drugs dispensed from community pharmacies within the province).

### 2.2. Patients

Patients were included in the study if they met the following criteria: were 18 years or older with de novo mUC or de novo unresected laUC, which was diagnosed between 2015 and 2019. The patient selection process identified all patients aged 18 years or older diagnosed with UC or mUC between 2015 and 2019 (*n* = 803); from which, those patients with de novo mUC or de novo unresected laUC were selected. The la/mUC stage was defined based on the depth of tumor invasion (primary tumor site and size), lymph node (primary and regional nodal extent of the tumor), and metastasis (absence or presence of metastases; TNM) stage using the most recent edition of the AJCC staging guidelines available at the time of initial diagnosis. No exclusion criteria were applied in this study.

#### Outcomes

For each line of systemic therapy, the treatment regimen was classified based on all systemic anti-cancer treatments received within the 14 days from initiating that line of therapy. The subsequent line of therapy was defined as the earliest of the following two events: receipt of any new systemic anti-cancer agent not within the initial regimen, or a treatment gap of more than 90 days between successive treatment dispensations. The end of therapy was defined as the earliest of the following three possible dates: date of the last cycle of the line of therapy plus 21 days, date of starting a subsequent line of therapy, or date of death or last known contact with the healthcare system. The primary outcome of interest was OS, defined as the time from the initiation of therapy (or from diagnosis for the entire population) until death from any cause. OS was also stratified by the therapy received, and according to the referral status and number of metastasis sites.

### 2.3. Statistical Analysis

Continuous study measures were reported descriptively with mean and standard deviation (SD). Frequencies and percentages were used to document categorical measures of interest, including the receipt of therapies at each line. For each treatment within each line of therapy, treatment duration was measured as the time from treatment initiation to time of treatment discontinuation, and reported as mean (SD) and median (interquartile range (IQR)). To account for individuals who were censored prior to the completion of therapy, the median time from treatment initiation until treatment cessation was also estimated using Kaplan–Meier analysis. Survival curves and median time-to-event were measured via the Kaplan–Meier method for treatment duration and OS. In situations where a patient was missing data required for a particular analysis, the individual was excluded from that specific analysis, but retained in all other analyses. Analyses were conducted using R version 4.2.1.

## 3. Results

### 3.1. Patient Characteristics

A total of 206 patients diagnosed with de novo unresectable la/mUC were identified and included in the study. The baseline characteristics of the study population are summarized in Table 1. The majority of patients had mUC (80.6%), and 46.6% of patients had one site of metastatic disease. The most frequently reported sites of metastases at diagnosis were lymph nodes (40.5%), bone (26.8%), and lung (21.5%).

### 3.2. Treatment Patterns

The majority of patients with la/mUC diagnosed from 2015 to 2019 did not receive any systemic therapy (65.0%, *n* = 134), as shown in Table 2. Of the patients with de novo la/mUC, 58.7% (*n* = 121/206) were referred to a medical oncologist, and 41.3% (85/206) were not referred. The mean (SD) age in the referred cohort was 70.0 (9.0) years, compared with 77.2 (10.1) years in the non-referred cohort. Among those referred to a medical oncologist, 57.9% (*n* = 70/121) initiated systemic first-line therapy. Of all patients, 20.9% (43/206) had surgery, and 35.0% (72/206) had radiation therapy.

The treatment patterns among patients with de novo la/mUC and the median duration of treatment are summarized in Table 3. A total of 72 patients (35.0% of the de novo cohort of 206 patients) received first-line systemic therapy; of these, 41.7% (30/72) and 40.3% (29/72) were treated with carboplatin-gemcitabine and cisplatin-gemcitabine, respectively. The median duration of first-line systemic treatment was 2.8 months (interquartile range (IQR), 3.3). Among patients who received first-line treatment, 48.6% (*n* = 35/72) were treated with subsequent second-line therapy; of those, the majority (65.7%; 23/35) received pembrolizumab. The median duration of second-line systemic treatment was 3.0 months (IQR, 3.3).

### 3.3. Overall Survival

**Figure 1:**
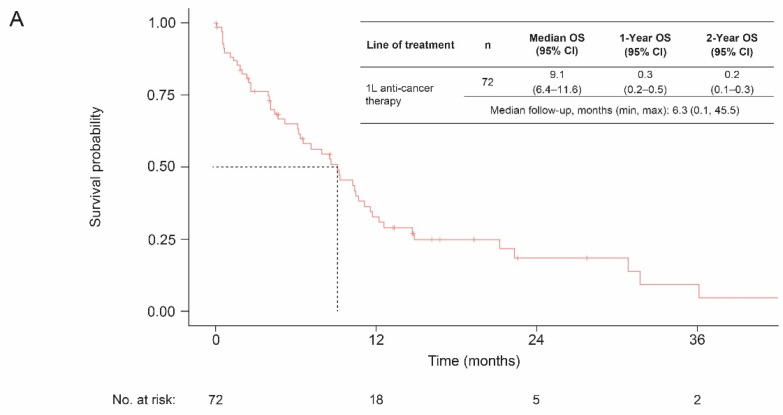

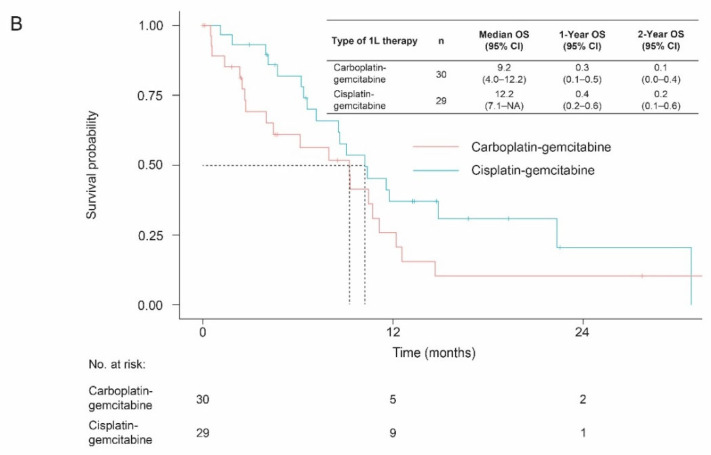
OS among patients with de novo la/mUC following initiation of 1L systemic therapy, (**A**) overall, and (**B**) by therapy received. Abbreviations: 1L, first line; CI, confidence interval; la/mUC, locally advanced or metastatic urothelial carcinoma; OS, overall survival.

In all patients with de novo la/mUC (*n* = 206), the median OS from diagnosis was 5.3 months (4.5–7.0). In the 72 patients who initiated first-line systemic therapy, the median follow-up was 6.3 months (range, 0.1–45.5) and the median OS from the time of initiation of first-line systemic therapy was 9.1 months (95% confidence interval (CI), 6.4–11.6) (Figure 1). When considering the type of first-line therapy received, the median OS among patients who received cisplatin-gemcitabine was higher than those who received carboplatin-gemcitabine (12.2 months (95%CI, 7.1–NA) vs. 9.2 months (95% CI, 4.0–12.2), respectively).

**Figure 2:**
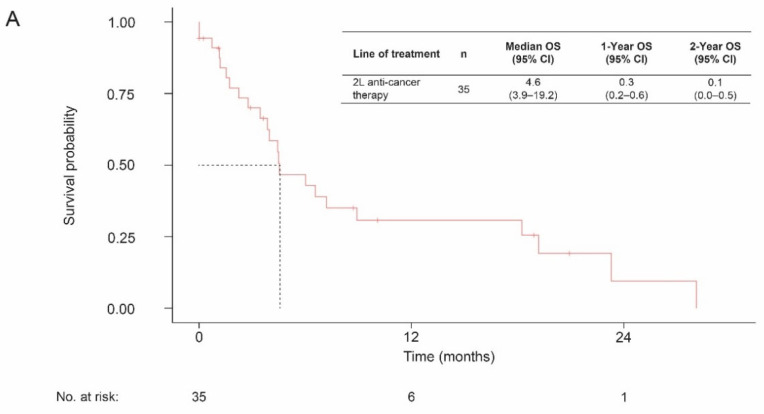

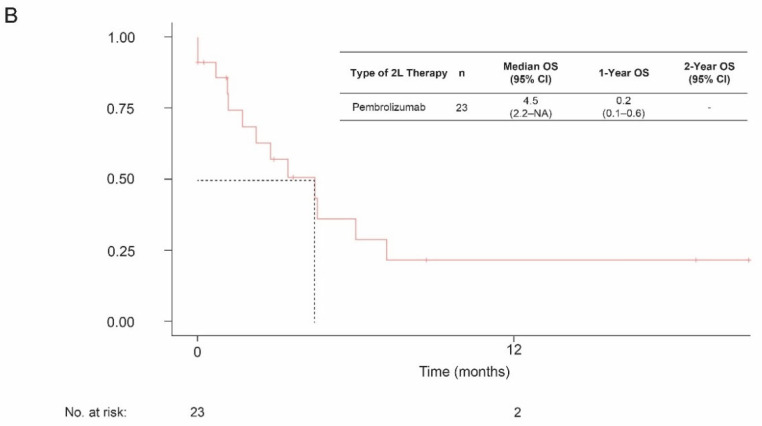
OS from start of second-line therapy among patients with de novo la/mUC following initiation of second-line systemic therapy, (**A**) overall, and (**B**) by therapy received. Abbreviations: 2L, second line; CI, confidence interval; la/mUC, locally advanced or metastatic urothelial carcinoma; NA, not available; OS, overall survival.

The median OS from the initiation of second-line systemic therapy was 4.6 months (95% CI, 3.9–19.2) (Figure 2). Patients who received pembrolizumab had a median OS of 4.5 months (95% CI, 2.2–NA).

**Figure 3:**
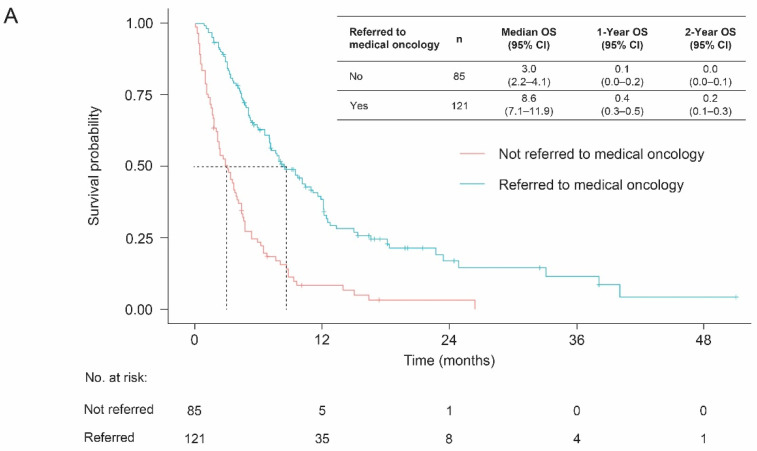

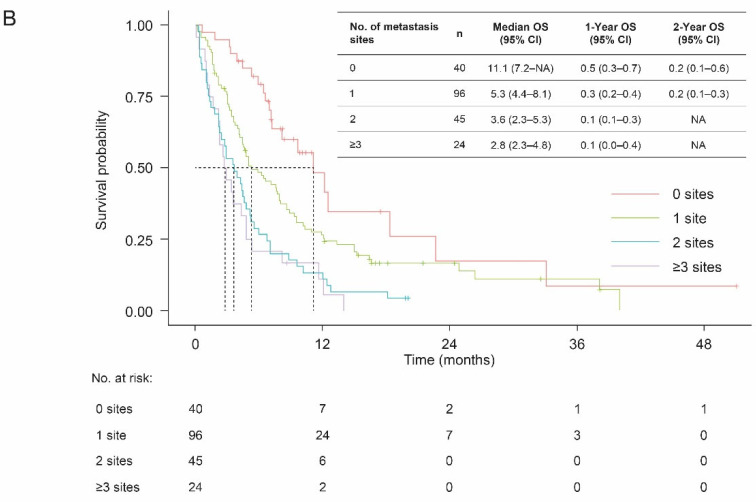
OS from diagnosis among patients with de novo la/mUC according to (**A**) referral status and (**B**) number of metastasis sites. Abbreviations: CI, confidence interval; la/mUC, locally advanced or metastatic urothelial carcinoma; NA, not available; OS, overall survival.

Among all patients with de novo la/mUC, those patients referred to medical oncology had a longer median OS than patients who were not referred (8.6 (95% CI, 7.1–11.9) vs. 3.0 (95% CI, 2.2–4.1) months, respectively) (Figure 3). Patients without metastases had a longer OS compared with those with multiple metastasis sites (the median OS for patients with 0 vs. ≥3 metastasis sites were 11.1 (95% CI, 7.2–NA) and 2.8 (95% CI, 2.3–4.8) months, respectively) (Figure 3).

## 4. Discussion

This retrospective longitudinal cohort study generated real-world evidence pertaining to treatment patterns and survival outcomes in a cohort of patients with de novo unresected la/mUC in Alberta, Canada, between 2015 and 2019. Overall, the uptake of systemic anti-cancer therapy was limited; 35.0% of patients received first-line therapy; of which, 48.6% subsequently received second-line therapy. Only 58.7% of patients were referred to a medical oncologist.

Among first-line-treated patients, most (82.0%) received platinum-based chemotherapy in the first-line setting (gemcitabine with either carboplatin (41.7%) or cisplatin (40.3%), whereas pembrolizumab immunotherapy was the most common second-line treatment (65.7%). Only half of the patients receiving first-line platinum-based therapy received cisplatin, and those patients had improved OS compared with those who received carboplatin (12.2 months vs. 9.2 months). These data are similar to a recent real-world study from the USA, in which approximately half of first-line-treated patients with la/mUC were eligible for cisplatin, and had a median OS of 14.4 months (compared with 8.6 months for patients who were ineligible for cisplatin) [17].

The majority of patients received platinum-based therapy during first-line treatment, which is consistent with a prior retrospective (2002–2016) analysis of 233 patients with mUC from Alberta, Canada, in which 55.8% of patients received first-line cisplatin-based therapy, and 37.3% received first-line carboplatin-based therapy [18]. These data highlight that though most patients receive platinum-based therapy consistent with the current standard of care, the outcomes are poor, demonstrating a need for novel platinum-free regimens in the first-line treatment setting in this patient population.

The median OS for patients treated in the first-line setting in this real-world population was similar to that noted in clinical trials. In the landmark clinical trial comparing cisplatin-gemcitabine with MVAC, the median OS was 13.8 months with first-line cisplatin-gemcitabine [19], compared with 12.2 months in this study. The median OS was 9.3 months with first-line carboplatin-gemcitabine in the EORTC Study 30986 [20], compared with 9.2 months in this study. Conversely, the OS in this analysis was notably shorter than in other real-world studies from Germany and the UK. A cohort analysis of patients with la/mUC in Germany (data from 2009–2016) reported a median OS of 16.1 months in the first-line setting, and 9.2 months in the second-line setting [21]. A study in the UK (data from 2003–2017) reported a median OS of 16.2 months among first-line-treated patients [22]. Although, in a more recent retrospective cohort analysis from the USA (data from 2016–2020), the median OS was 11.0 months [17].

In contrast with the first-line outcomes noted in this patient population, the median OS with second-line pembrolizumab was shorter in this study (4.5 months) than the 10.3 months reported in the KEYNOTE-045 clinical trial [23]. The median duration of second-line therapy in this study was 3.0 months, slightly shorter than the 3.5-month median duration of therapy in KEYNOTE-045 [23]. However, the real-world population in this study may not be directly comparable to those in the above clinical trials. The relatively short duration of therapy in this study (the median duration of first-line therapy was 2.8 months) suggests that some patients progressed at their first scan.

The treatment landscape for patients with la/mUC has continued to evolve since the time of this study (from 2015–2019). Immunotherapies including immune-checkpoint inhibitors such as avelumab, approved for maintenance therapy by Health Canada in 2021 [12], and pembrolizumab, approved in the second-line setting [14], are now more widely integrated into clinical practice. Not all patients respond to immune-checkpoint inhibitors, and the identification of those patients who are more likely to respond is an important area of ongoing research [24,25]. During the period of this study, the absence of reimbursement for second-line immunotherapies may have contributed to the low rate of referrals to medical oncologists for those patients who were thought to be unfit for chemotherapy. Of all patients in this study, only 58.7% were referred to a medical oncologist; among these, only 57.9% of patients initiated first-line therapy. Patients who were not referred to a medical oncologist were typically older and experienced poorer outcomes than patients who were referred.

Other novel therapies have also contributed to improvements in survival in clinical trials, and are currently being implemented in real-world practice. In the phase III EV-301 study, enfortumab vedotin, approved by Health Canada in 2021 [15], demonstrated an improvement in OS compared with standard chemotherapy in patients with la/mUC previously treated with platinum-based chemotherapy and a PD-1/L1 inhibitor (12.9 vs. 9.0 months; hazard ratio for death (95% CI), 0.70 (0.56–0.89); *p* = 0.001) [26]. In 2019, erdafitinib received conditional approval from Health Canada based on objective response rates in patients with susceptible fibroblast growth factor receptor(*FGFR*)2 or *FGFR3* genetic alterations whose disease had progressed during or following a prior line of chemotherapy [16]. Approval is pending completion of ongoing phase III clinical trials [27]. In the future, treatment patterns are likely to change further with new therapies now available or under investigation.

### Limitations

The population in this analysis was small (*n* = 206), and patients were identified from only one province in Canada. Therefore, the findings might not be generalizable to the overall population of patients with la/mUC in Canada. Furthermore, direct statistical comparisons between sub-groups were not conducted, given the relatively small size of the populations. It was noted that patients who were not referred to a medical oncologist were older, with more advanced disease and poorer outcomes, than patients who were referred. Though the inferior outcomes may have been attributable to patients with advanced disease deciding not to pursue treatment, the available data do not allow firm conclusions. As the registry databases used for this study capture cases at their first entry, it was not possible to ascertain recurrent or relapsed cases, and, as a result, the analysis could only include patients who presented with de novo la/mUC. In addition, the latest available data were from 2019, so more recent changes in practices such as the reimbursement for, and wider use of, some immunotherapies since 2020 are not represented in this analysis. Nevertheless, the results from this study add to the limited real-world evidence currently available on treatment patterns and clinical outcomes in la/mUC prior to the integration of novel therapies into the standard treatment algorithm.

## 5. Conclusions

These results present a snapshot in time of the de novo la/mUC population, including treatment patterns prior to the reimbursement, and widespread use, of novel therapies such as pembrolizumab, avelumab, enfortumab vedotin, and erdafitinib. Many patients were not referred to a medical oncologist, and the large majority of patients (65.0%) did not receive any first-line systemic therapy, and among those that did, less than half received subsequent second-line therapy. Overall, survival rates were poor in patients with la/mUC. Our study highlights the importance of referral to a medical oncologist and the ongoing need for effective therapies in these patients. Future work will evaluate outcomes for patients with la/mUC after the widespread use and availability of novel therapies.

## Figures and Tables

**Table 1:** Baseline characteristics of patients with de novo la/mUC.

Variable		De Novo Cohort (*N* = 206)
Age	Mean (SD), years	73.0 (10.1)
	<60 years, *n* (%)	45 (21.8)
	≥60 years, *n* (%)	161 (78.2)
Sex, *n* (%)	Female	43 (20.9)
	Male	163 (79.1)
Year of diagnosis, *n* (%) [median follow-up, months]	2015	32 (15.5) [4.0]
	2016	31 (15.0) [6.0]
	2017	43 (20.9) [4.4]
	2018	33 (16.0) [4.6]
	2019	67 (32.5) [5.9]
AJCC TNM stage at diagnosis, *n* (%)	Unresected locally advanced	40 (19.4)
	Metastatic UC	166 (80.6)
Disease histology, *n* (%)	TCC	206 (100)
No. of metastasis sites at diagnosis, *n* (%)	0	40 (19.4)
	1	96 (46.6)
	2	45 (21.8)
	≥3	24 (11.7)
	Missing	1 (0.5)
Sites of metastases at diagnosis ^a^, *n* (%)	Lymph nodes	83 (40.5)
	Bone	55 (26.8)
	Lung	44 (21.5)
	Hepatic	36 (17.6)
	Peritoneum	20 (9.8)
	Adrenals	<10
	Brain	<10
	Other	18 (8.8)

^a^ One patient was excluded from the analyses due to missing data on metastasis sites; patients may have more than one metastasis in more than one site. Abbreviations: AJCC, American Joint Committee on Cancer; TCC, transitional cell carcinoma; TNM, tumor node metastasis; UC, urothelial carcinoma.

**Table 2:** Numbers of incident cases of patients with de novo la/mUC who received no systemic therapy, and of patients with de novo la/mUC who received 1L systemic therapy.

	Overall, *n* (%)	Year of Diagnosis, *n* (%) ^a^			
		2015–2016	2017	2018	2019
No systemic therapy	134 (65.0)	41 (65.1)	30 (69.8)	22 (66.7)	41 (61.2)
1L systemic therapy	72 (35.0)	22 (34.9)	13 (30.2)	11 (33.3)	26 (38.8)

^a ^ Percentages are of all cases for each year(s). Abbreviations: 1L, first line; la/mUC, locally advanced or metastatic urothelial carcinoma.

**Table 3:** Types of treatments and duration of therapy among patients with de novo la/mUC.

Treatment	N (%)	Duration of Treatment, Months		
		Mean (SD)	Median [25th–75th Percentile]	Median (Kaplan-Meier)
**1L anti-cancer therapies**	**72 (100.0)**	**3.2 (2.3)**	**2.8 [1.1–4.4]**	**3.6**
Carboplatin-gemcitabine	30 (41.7)	3.5 (2.6)	3.0 [1.1–4.6]	4.2
Cisplatin-gemcitabine	29 (40.3)	3.4 (1.9)	3.1 [1.9–4.4]	3.5
Other ^a^	13 (18.1)	-	-	-
**2L anti-cancer therapies**	**35 (100.0)**	**4.2 (4.7)**	**3.0 [1.6–5.0]**	**4.4**
Pembrolizumab	23 (65.7)	4.4 (5.4)	3.0 [1.4–5.0]	5.4
Other ^a^	12 (34.3)	-	-	-

^a^ Cells were suppressed due to cell sizes < 10 and due to the ability to infer certain strata < 10 based on marginal counts. Abbreviations: 1L, first line; 2 L, second line; SD, standard deviation.

## Data Availability

Data presented in this study are aggregate-level data, individual-level data are not publicly available due to Canadian data privacy laws governing personal health information.
